# Supplementary material for: Oxygen provision to severely ill COVID-19 patients at the peak of the 2020 pandemic in a Swedish district hospital
Source: PLoS One. 2022 Jan 20;17(1):e0249984. doi: 10.1371/journal.pone.0249984 (PMC8775206; doi:10.1371/journal.pone.0249984)
Supplement: S1 Table — (DOCX) [file pone.0249984.s001.docx]

**S1 Table Patient characteristics and outcomes**

|  | **Patients admitted to the wards who never received oxygen treatment**  **% (n/N), unless otherwise stated** | **Patients initially admitted to the wards and who were later transferred to ICU**  **% (n/N), unless otherwise stated** | |
| --- | --- | --- | --- |
| **Age (years), median (IQR)** | 60 (52-73) | 61 (56-70) | |
| **Female** | 52% (25/48) | 22% (4/18) | |
| **Diagnosis of COVID-19 confirmed by PCR** | 81% (39/48) | 100% (18/18) | |
| **BMI, ≥30** | 23% (7/30) | 36% (4/11) | |
| **CACI ≥4** | 35% (17/48) | 17% (3/18) | |
| **No-ICU-decision documented , n (%)** | 31% (14/48) | 11% (2/18) | |
| *Red NEWS-2 [1] parameter on first measurements of vital signs* | | |  |
| **SpO2** (≤91%) | 2.1% (1/48) | 56% (10/18) | |
| **Respiratory rate**  (≤8 or ≥25 breaths/min**)** | 24% (11/45) | 44% (8/18) | |
| **Heart rate**  (≤40 or **≥**131 beats/min) | 2.1% (1/48) | 0% (0/18) | |
| **Systolic blood pressure**  (≤90 or ≥220 mmHg) | 2.1% (1/48) | 0% (0/18) | |
| **Consciousness**  (Non-alert) | 6.4% (3/47) | 0% (0/18) | |
| **Temperature**  (≤35.0 or ≥39.1°C) | 8.3% (4/48) | 44% (8/18) | |
| *Treatments during hospital-stay* | | | |
| **Antibiotics** | 56% (27/48) | 100% (18/18) | |
| **Chloroquine** | 6.2% (3/48) | 28% (5/18) | |
| **Anticoagulants** | 40% (19/48) | 94% (17/18) | |
| *Outcomes* |  |  | |
| **Length of stay (days), median (IQR)** | 1.9 (1.1-3.1) | 24 (9.5-31) | |
| **Transfer to another department** | 4% (2/48) | 50% (9/18) | |
| **Dead in-hospital** | 0% (0/48) | 22% (4/18) | |
| **Dead at 60 days** | 0% (0/48) | 22% (4/18) | |

*Abbreviations: PCR: polymerase chain reaction, BMI: body mass index, CACI: Charlson´s age adjusted comorbidity score, ICU: intensive care unit, SpO2: peripheral oxygen saturation

** For two patients the initial no-ICU decisions were changed after time and the patients were transferred to ICU.

**^1^** Royal College of Physicians. National Early Warning Score (NEWS) 2. Standardising the assessment of acute-illness severity in the NHS. Updated report of a working party. [Internet]. London: RCP; 2017 [cited 2021 Jan 6]. Available from: https://www.rcplondon.ac.uk/projects/outputs/national-early-warning-score-news-2
