# Supplementary material for: Oxygen provision to severely ill COVID-19 patients at the peak of the 2020 pandemic in a Swedish district hospital
Source: PLoS One. 2022 Jan 20;17(1):e0249984. doi: 10.1371/journal.pone.0249984 (PMC8775206; doi:10.1371/journal.pone.0249984)
Supplement: S2 Table — (DOCX) [file pone.0249984.s002.docx]

**S2 Table Clinical progression score for all admitted patients^2^**

| **Descriptor** | **Score** | **All admitted patients (n=206)** | **All admitted patients aged <70 (n=120)** | **All admitted patients with no-ICU-decision (n=77)** |
| --- | --- | --- | --- | --- |
| Hospitalized without oxygen | 4 | 23% (48/206) | 29% (35/120) | 19% (15/77) |
| Hospitalized with oxygen nasal spongs or mask | 5 | 43% (88/206) | 49% (59/120) | 27% (21/77) |
| Hospitalised; oxygen by NIV or high flow | 6 | 1% (2/206) | 2% (2/120) | 0% (0/77) |
| Intubation and mechanical ventilator | 7 to 9 | 10% (20/206) | 12% (14/120) | 1% (1/77) |
| Dead after 60 days | 10 | 23% (48/206) | 8.3% (10/120) | 52% (40/77) |

^2^ Clinical progression score, adapted from Marshall JC, Murthy S, Diaz J, Adhikari NK, Angus DC, Arabi YM, et al. A minimal common outcome measure set for COVID-19 clinical research. Lancet Infect Dis. 2020 Aug 1;20(8):e192–7.

*Initial no-ICU decision changed after time and the patient transferred to ICU.
