# Supplementary material for: Oxygen provision to severely ill COVID-19 patients at the peak of the 2020 pandemic in a Swedish district hospital
Source: PLoS One. 2022 Jan 20;17(1):e0249984. doi: 10.1371/journal.pone.0249984 (PMC8775206; doi:10.1371/journal.pone.0249984)
Supplement: S3 Table — (DOCX) [file pone.0249984.s003.docx]

**S3 Table Oxygen provision in the wards to patients that were later transferred to ICU**

|  | Patients initially admitted to the wards that later transferred to ICU (N=18) |
| --- | --- |
| Days on oxygen treatment before transfer to ICU, median (IQR) | 1.8 (0.32-5.0) |
| Oxygen flow to patients during oxygen therapy (l/min) |  |
| - Mean (SD) | 7.5 (3.4) |
| - Median (IQR) | 7.5 (4.5-9.3) |
| Oxygen flow to patients during time in the ward (l/min) |  |
| - Mean (SD) | 6.7 (4.0) |
| - Median (IQR) | 7.0 (3.3-8.5) |
| Total volume of oxygen provided per patient admission (l) |  |
| - Mean (SD) | 34,000 (51,000) |
| - Median (IQR) | 13,000 (4,100-59,000) |
