# Supplementary material for: Oxygen provision to severely ill COVID-19 patients at the peak of the 2020 pandemic in a Swedish district hospital
Source: PLoS One. 2022 Jan 20;17(1):e0249984. doi: 10.1371/journal.pone.0249984 (PMC8775206; doi:10.1371/journal.pone.0249984)
Supplement: Attachment — Submitted filename: Response to Reviewers.docx [file pone.0249984.s004.docx]

| **Reviewer’s comment** | **Our response** |
| --- | --- |
| REVIEWER #1  The study does seem to be interesting especially in this pandemic where a lot of countries are facing paucity of oxygen. Few comments though. | Thank you! |
| 1)Could the authors please elaborate on the CACI scoring system for the benefit of the readers? | The following is added in rows 108-119:  Charlson´s age adjusted comorbidity score (CACI) was calculated for all patients and noted in the chart for each patient. The CACI score (from 0 to 37) predicts 10-year-survival in patients, accounting for age and multiple comorbidities For example a CACI-score of 4 points estimates 53% 10-year-survival; 0 points 98% and ≥7 points 0% 10-year-survival(26,27)**.** |
| 2)Did any of the patients have any underlying pulmonary disease like Bronchial asthma,COPD, structural lung disease like bronchiectasis? | We did not collect data on pre-existing diagnoses in detail but chose to use the CACI score as an indicator of the total burden of underlying illness for each patient. |
| 3)Did any of them have underlying cardiac issues which could have aggravated the hypoxemia? | Please, see above. |
| 4)How was 02 administered ,nasal prongs,face masks,Venturi masks etc? | We have added this description on O2-administration in rows 92-94:  “(…)oxygen therapy through nasal prongs (<5l/min) or face masks (≥5l/min) (or, when indicated, with reservoir bags) was adjusted to maintain SpO2 within an individualized target range.” |
| 5)What was the source of 02 supply-concentrators, cylinders,central supply source? | All oxygen came from a central supply source. We have added this in rows 90-91:  ”Piped oxygen was provided from a central supply source to the wards.” |
| REVIEWER#2: Thank you for allowing me to review " Oxygen provision to severely ill COVID-19 patients at the peak of the 2020 pandemic in a Swedish district hospital" by Anna Hvarfner et al. The authors retrospectively surveyed oxygen flow-rate and total volume of oxygen in severely ill COVID-19-patients. The mean oxygen flow-rate was 3.0 l/min, and lower than previously estimated. This is an interesting study. Although oxygen flow-rate might vary according to targeted SpO2, the authors stated about it.  My comments are as follows. | Thank you! |
| “The WHO estimates the average flow-rate of oxygen to severely ill COVID 19-patients (referring to those requiring oxygen but not intensive care unit treatment) to be 10 l/min.” I couldn't find this description in reference 22. Where is it listed? | We agree that this is not very clearly stated in the reference. However in the first paragraph of the “COVID-19 and oxygen” section there is a description of different severity stages of COVID-19 stating that “…about 15 % of them have severe illness requiring oxygen therapy, and 5% will be critically ill requiring intensive care unit treatment.” Further down in table 1 there is a presentation of the oxygen needs for these two groups (for severe patients 10 L/min). |
| There seems to some missing data in table 1. How do you handle missing values? | We used pairwise deletion, i.e.. including all available cases per variable in the descriptive data in Table 1. We have added a sentence clarifying this in rows 181-182.  “Missing descriptive data were delt with by pairwise deletion, i.e. all available data per variable was included in analysis.” |
|  |  |

| Journal Requirements | Our actions |
| --- | --- |
| 1.Please ensure that your manuscript meets PLOS ONE's style requirements, including those for file naming. The PLOS ONE style templates can be found at  https://journals.plos.org/plosone/s/file?id=wjVg/PLOSOne_formatting_sample_main_body.pdf and  <https://journals.plos.org/plosone/s/file?id=ba62/PLOSOne_formatting_sample_title_authors_affiliations.pdf> | To the best of our ability the title page, the main body, and the supporting information have been adjusted. The figure file is relabeled. |
| 2.Please review your reference list to ensure that it is complete and correct. If you have cited papers that have been retracted, please include the rationale for doing so in the manuscript text, or remove these references and replace them with relevant current references. Any changes to the reference list should be mentioned in the rebuttal letter that accompanies your revised manuscript. If you need to cite a retracted article, indicate the article’s retracted status in the References list and also include a citation and full reference for the retraction notice. | The reference list is reviewed. We have added one reference to a recently published study of the unmet need of oxygen treatment in critical illness (15). |
| 3. Thank you for stating the following in the Competing Interests section:  [I have read the journal's policy and the authors of this manuscript have following competing interests: Dr. Baker reports a grant and personal fees from Wellcome Trust, and personal fees from UNICEF and the World Bank, all outside the submitted work.].  Please confirm that this does not alter your adherence to all PLOS ONE policies on sharing data and materials, by including the following statement: "This does not alter our adherence to  PLOS ONE policies on sharing data and materials.” (as detailed online in our guide for authors [http://journals.plos.org/plosone/s/competing-interests](about:blank)).  If there are restrictions on sharing of data and/or materials, please state these. Please note that we cannot proceed with consideration of your article until this information has been declared.  Please include your updated Competing Interests statement in your cover letter; we will change the online submission form on your behalf.  Please know it is PLOS ONE policy for corresponding authors to declare, on behalf of all authors, all potential competing interests for the purposes of transparency. PLOS defines a competing interest as anything that interferes with, or could reasonably be perceived as interfering with, the full and objective presentation, peer review, editorial decision-making, or publication of research or non-research articles submitted to one of the journals. Competing interests can be financial or non-financial, professional, or personal. Competing interests can arise in relationship to an organization or another person. Please follow this link to our website for more details on competing interests: [http://journals.plos.org/plosone/s/competing-interests](about:blank) | - Done, see cover letter and here:   I have read the journal's policy and the authors of this manuscript have the following competing interests: Dr. Baker reports a grant and personal fees from Wellcome Trust, and personal fees from UNICEF and the World Bank, all outside the submitted work. This does not alter our adherence to  PLOS ONE policies on sharing data and materials. The other authors declare no competing interests. |
| 4. We note that you have indicated that data from this study are available upon request. PLOS only allows data to be available upon request if there are legal or ethical restrictions on sharing data publicly. For information on unacceptable data access restrictions, please see http://journals.plos.org/plosone/s/data-availability#loc-unacceptable-data-access-restrictions.  In your revised cover letter, please address the following prompts:  a) If there are ethical or legal restrictions on sharing a de-identified data set, please explain them in detail (e.g., data contain potentially identifying or sensitive patient information) and who has imposed them (e.g., an ethics committee). Please also provide contact information for a data access committee, ethics committee, or other institutional body to which data requests may be sent.  b) If there are no restrictions, please upload the minimal anonymized data set necessary to replicate your study findings as either Supporting Information files or to a stable, public repository and provide us with the relevant URLs, DOIs, or accession numbers. Please see http://www.bmj.com/content/340/bmj.c181.long for guidelines on how to de-identify and prepare clinical data for publication. For a list of acceptable repositories, please see http://journals.plos.org/plosone/s/data-availability#loc-recommended-repositories.  We will update your Data Availability statement on your behalf to reflect the information you provide. | Updated data availability statement added in cover letter and here:  Data cannot be shared publicly because of ethical and legal restrictions linked to the content of sensitive patient information. Anonymized data can be requested via Carl Otto Schell (Department of Internal medicine, Nyköping hospital, S-61185 Nyköping, Sweden; [carl.schell@ki.se](mailto:carl.schell@ki.se)) or via Jesper Sperber (Head of department, Anaesthesia and Intensive care, Eskilstuna hospital, S-633 49 Eskilstuna, Sweden, [Jesper.sperber@regionsormland.se](mailto:Jesper.sperber@regionsormland.se)) for researchers who provide a justified and reasonable research plan for which the data are required. |
| 5. Please include captions for your Supporting Information files at the end of your manuscript, and update any in-text citations to match accordingly. Please see our Supporting Information guidelines for more information: [http://journals.plos.org/plosone/s/supporting-information](about:blank). | Done |
|  |  |
