# Supplementary material for: Oxygen provision to severely ill COVID-19 patients at the peak of the 2020 pandemic in a Swedish district hospital
Source: PLoS One. 2022 Jan 20;17(1):e0249984. doi: 10.1371/journal.pone.0249984 (PMC8775206; doi:10.1371/journal.pone.0249984)
Supplement: Attachment — Submitted filename: Response to Reviewers.docx [file pone.0249984.s005.docx]

## Reviewer’s comment

Reviewer #2: Thank you for allowing me to review " Oxygen provision to severely ill COVID-19 patients at the peak of the 2020 pandemic in a Swedish district hospital" by Anna Hvarfner et al.
The authors addressed the reviewer’s comments on a point-by-point basis.

My comments are as follows.
*“- We agree that this is not very clearly stated in the reference. However in the first
paragraph of the “COVID-19 and oxygen” section there is a description of different
severity stages of COVID-19 stating that “…about 15 % of them have severe illness
requiring oxygen therapy, and 5% will be critically ill requiring intensive care unit
treatment.” Further down in table 1 there is a presentation of the oxygen needs for
these two groups (for severe patients 10 L/min).”*

Sorry, I could not find the authors description in reference 23. Where is it listed? I could not find table 1.

## Our response

***We are sorry that our previous response was not clear. Reference 23 contains the information in the first paragraph of the “COVID-19 and oxygen” section on the first page and in Table 1 on the second page. We highlight the relevant parts in the screenshots below.***
